# Lessons and implications from a mass immunization campaign in squatter settlements of Karachi, Pakistan: an experience from a cluster-randomized double-blinded vaccine trial [NCT00125047]

**DOI:** 10.1186/1745-6215-7-17

**Published:** 2006-05-25

**Authors:** Mohammad Imran Khan, Rion Leon Ochiai, Hasan Bin Hamza, Shah Muhammad Sahito, Muhammad Atif Habib, Sajid Bashir Soofi, Naveed Sarwar Bhutto, Shahid Rasool, Mahesh K Puri, Mohammad Ali, Shafi Mohammad Wasan, Mohammad Jawed Khan, Remon Abu-Elyazeed, Bernard Ivanoff, Claudia M Galindo, Tikki Pang, Allan Donner, Lorenz von Seidlein, Camilo J Acosta, John D Clemens, Shaikh Qamaruddin Nizami, Zulfiqar A Bhutta

**Affiliations:** 1 Department of Pediatrics, Aga Khan University, Karachi, Pakistan; 2 International Vaccine Institute, Seoul, Korea; 3 Department of Family Medicine, Aga Khan University, Karachi, Pakistan; 4 US NAMRU 3, Cairo, Egypt; 5 GlaxoSmithKline Biologicals, Singapore; 6 Vaccines and Other Biologicals, World Health Organization, Geneva, Switzerland; 7 Research Policy and Cooperation, World Health Organization, Geneva, Switzerland; 8 University of Western Ontario, Canada

## Abstract

**Objective:**

To determine the safety and logistic feasibility of a mass immunization strategy outside the local immunization program in the pediatric population of urban squatter settlements in Karachi, Pakistan.

**Methods:**

A cluster-randomized double blind preventive trial was launched in August 2003 in 60 geographic clusters covering 21,059 children ages 2 to 16 years. After consent was obtained from parents or guardians, eligible children were immunized parenterally at vaccination posts in each cluster with Vi polysaccharide or hepatitis A vaccine. Safety, logistics, and standards were monitored and documented.

**Results:**

The vaccine coverage of the population was 74% and was higher in those under age 10 years. No life-threatening serious adverse events were reported. Adverse events occurred in less than 1% of all vaccine recipients and the main reactions reported were fever and local pain. The proportion of adverse events in Vi polysaccharide and hepatitis A recipients will not be known until the end of the trial when the code is broken. Throughout the vaccination campaign safe injection practices were maintained and the cold chain was not interrupted. Mass vaccination in slums had good acceptance. Because populations in such areas are highly mobile, settlement conditions could affect coverage. Systemic reactions were uncommon and local reactions were mild and transient. Close community involvement was pivotal for information dissemination and immunization coverage.

**Conclusion:**

This vaccine strategy described together with other information that will soon be available in the area (cost/effectiveness, vaccine delivery costs, etc) will make typhoid fever control become a reality in the near future.

## Background

Despite major breakthroughs in the development of new vaccines over the past two decades, the gap in access to vaccines between wealthy and poorer countries has widened. As a result, immunization schedules offer more vaccines in high-income countries than in those with low income [1]. Children in low-income countries are also at a disadvantage because vaccine research and development agendas are tailored to the needs of developed countries. The focus the International Vaccine Institute typhoid fever program is to enable people at risk to get access to the vaccines and decrease the burden of typhoid fever [2]. The program is being conducted in five urban slums of Indonesia, China, Vietnam, India and Pakistan.

Two typhoid fever vaccines, Ty21a and Vi polysaccharide (PS) are currently licensed for use. A recent Cochrane review [3] on typhoid fever vaccines found that the two vaccines i.e. Ty 21 and Vi have similar results with Vi having an advantage of heat stability and single dose regimen. ViPS was thus chosen for use in the DOMI trial as it would suit the public health program for immunization in the countries of south east Asia. The use of Vi requiring a single, injectable dose was thought to be logistically easier than use of Ty21 which requires three doses.

In Pakistan, DOMI program aims to introduce an available, affordable Vi polysaccharide vaccine [4,5] for children 2 to 16 years of age living in urban low socio economic settings. The study setting has a high typhoid fever burden and treatment is increasingly costly [6] and difficult due to high drug resistance[7,8]. Vi PS vaccine, which has moderate efficacy (64–77%), seems to be an immediate and affordable option for impoverished populations exposed to typhoid fever[9]. Unfortunately, there is no evidence about its cost-effectiveness and no delivery strategy has been envisaged that would enable policymakers to make rational decisions about the use of this vaccine as a public health tool.

To determine the effectiveness of Vi PS in reducing typhoid fever burden in slum areas and the cost-effectiveness of the vaccine, DOMI investigators designed a cluster-randomized double blind trial [2]. Given the apparent immunological limitation of PS vaccines in early age groups [10] and the fact that the high-risk group in Karachi is the entire pediatric population, the local Expanded Program of Immunization (EPI), which usually reaches children under age 5 years, is unlikely to be the best delivery structure. Also, national reported immunization coverage rates for Pakistan have been very variable (range of 60–90%) since 1998 [11]. In addition to a lack of resources, other documented reasons for low vaccine coverage in Pakistan include lack of awareness of need, mothers unable to attend the vaccine posts, and inconvenient immunization sites [12-14] For these reasons, the DOMI program decided to implement the mass immunization campaign outside the EPI delivery system. Here we report initial results from this mass vaccination in two slums in Karachi, Pakistan.

## Methods

### Study site

The vaccination campaign was carried out in two adjacent squatter settlements, Sultanabad and Hijrat Colony, in Karachi. The population is a mix of Punjabi-Pathan ethnic groups from northern Pakistan. The total population of the two areas is 53,738 (project census 2003), with 21,059 children (aged 2–16 years) in the study target pediatric population. There are 8,278 households in the combined settlement areas of 0.54 km^2^. Most health-care is provided by the private sector through small clinics. In the last 7 years the Department of Pediatrics of Aga Khan University [7] has rendered free clinical services to the pediatric population through health centers, one in each study area. These are staffed with research medical officers (RMO), health assistants, field supervisors, and community health workers (CHW). The study area is considered to be a high endemic area for typhoid fever, especially among children [6,15].

### Sample size

A total of approximately 24,710 children are needed in order to have 80% power to detect a 50% vaccine protection at a 5% level of significance. Using Hayes and Bennet formula [16], this sample size calculation assumes a minimum cumulative typhoid incidence of 2.8 per 1000 (during 2 years), assuming alpha = 0.05, minimum power 0.8 (= 1-beta) to achieve a significant difference, Protective Efficacy (PE) of 0.5 for 2 years, between cluster coefficient of variation (CV) below 0.5 an average cluster size of 580.

### Study design

The cluster-randomized design employed by the trial mimics the way Vi vaccine would be delivered under a public health program in Pakistan. The study area, Sultanabad and Hijrat Colony were divided into 28 and 32 geographic clusters (a group of adjacent households), respectively (figure 2). Cluster sizes varied from 162 to 653 children (2–16 years of age) with an average of 350. The unit of randomization (clusters) was stratified by slum (Hijrat or Sultanabad) and cluster size (large of small). The eligible population was children aged 2–16 years who were included in the project census and whose parents/guardians gave consent to participate. These 60 geographic areas (clusters, 32 in Hijrat colony and 28 in Sultanabad) were randomly allocated to Vi PS vaccine (Typherix^®^) or the active control hepatitis A vaccine (Havrix^®^). Randomization was done by an expert statistician who was independent of disease surveillance activities in the study setting. The local investigators were not aware which vaccine was assigned codes (C & M). Labeling of the vaccine was done by the vaccine producer in Rixensart, Belgium. The randomization sequence was not changed at any level once it was initially generated. Each team worked with the single code throughout the campaign to minimize the risk of mix-ups. The schedule of cluster visits were arranged in such a way that the number of vaccine in both groups given per day was approximately equal. Continuous supervisory visits by supervisory team and external monitors ensured that all procedures were followed according to the protocol.

**Figure 1:**
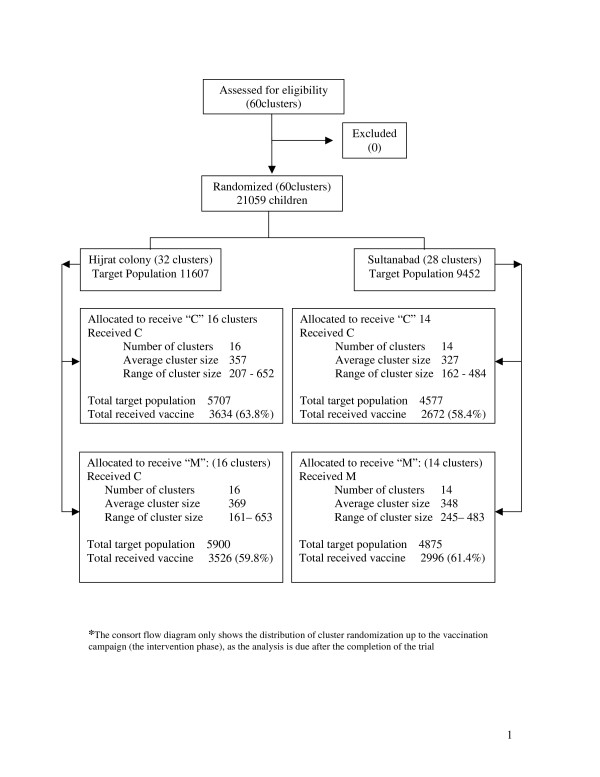
Distribution of target population and vaccination coverage Vi trial in Karachi Pakistan.

### The vaccines

Both vaccines, ViPS and HAV, are licensed in Pakistan and were donated by GlaxoSmithKline (GSK). Similar individual-dose-syringe vaccines were labelled with information on: the batch number, expiry date, route of administration and code (C or M). The identification of codes was kept with the Data Safety and Monitoring Board (DSMB). Each 0.5 ml dose of Typherix^® ^contained 25 micrograms of the Vi PS of *S*. Typhi. Each 0.5 ml of pediatric dose of Havrix^® ^vaccine consists of not less than 720 units of viral antigen, adsorbed on 0.25 mg aluminum hydroxide. The Havrix^® ^dosage consisted of a primary course and a booster that will be administered after the study ends (year 2). Both vaccines are for intramuscular injection only. Both groups will ultimately receive the benefits of the Vi vaccine as well as the HAV vaccine as a cross-over vaccination is planned at the end of the surveillance period.

In case of an adverse event the physician in charge examined the vaccine recipients and assess the severity of the event. In case of an event requiring hospitalization, the clinical monitor was notified who visited the patient and assessed the need of breaking the code after managing the case.

This project was approved by the AKU (Karachi) ethical committee, the Institutional Review Board of the International Vaccine Institute (IVI), Seoul, and the World Health Organization (WHO) ethical committee. A Data Safety and Monitoring Board (DSMB) was established for the audit, protocol review and take a decision on breaking the codes in case the breaking of code was deemed necessary due to an adverse event.

### Information dissemination and consent

Information dissemination started 12 weeks before the campaign. Sessions at street level were conducted by Research Medical Officers (RMO) or trained female Community Health Workers (CHW), as appropriate, and focused on the importance of immunization against typhoid fever and other control measures. More intense promotion of the campaign began in June 2003, 4 weeks prior to the campaign, by a team of RMOs and social scientists, who conducted meetings with community and religious leaders, members of local government bodies, and street representatives. Information leaflets were distributed and announcements were made at local mosques. Suitable areas for vaccination posts in each cluster were identified. The day before the vaccination date, households were visited and given formal invitation letters that included the site, date, and time for vaccination. During the campaign repeated visits were made by CHWs to remind and motivate those targeted for vaccination.

Parents and guardians visiting vaccination posts on the day of immunization were given information about the nature of the trial, expected risks and benefits, and procedural details as part of the informed consent. Trained project personnel provided this information. Upon agreeing to participate, a thumbprint was affixed on the vaccination record book together with the signature of a witness.

### Training and logistics

Vaccination teams received intensive training that focused on the trial's primary objectives, public health implications, blinding, cold chain maintenance, adverse events (AE), and community mobilization. Good Clinical Practices (GCPs) were emphasized at every step of training and monitored throughout the campaign. An adverse event was defined as a medical incident that takes place after vaccination, causes concern and is believed to be caused by the immunization. Continuous feedback meetings were conducted at intervals throughout the campaign.

Ten vaccination teams were employed consisting each of 1 physician, 1 vaccinator, 1 recorder, and 2 or 3 assistants. All were recruited specifically for the campaign and vaccinators were hired locally from the study sites. Physicians (team leaders) were responsible for the clinical and logistical components of their respective group. Supervisors (4) were in charge of two or three teams. External observers (2) monitored and documented key aspects (consent process, standards, safety, and cold chain) on designated forms. Seven community members assisted the campaign. Most of the vaccination posts were rooms or entire homes provided by residents and agreed upon by community leaders. Additionally, four local social scientists developed and organized the community awareness of the campaign. AKU drivers (5) assisted with transportation of supplies.

Vaccines were transported from Belgium to a local warehouse in Karachi. Recommended storage temperature of both vaccines is +2°C to +8°C. The temperature was monitored by one or more of the following methods: conventional thermometer, maximum-minimum thermometer, visual indicators of low temperature (FreezeWatch™, 3 M, USA), wheel recorders, and automated battery-powered devices (COX Technology, Sensitech, Inc., Beverly, MA, USA). Vaccines were stored in several sites: the central warehouse, the AKU warehouse, a field site logistics center, and cool boxes at vaccination posts. At the logistics center an officer distributed the vaccine on a daily basis to each vaccination team. Temperature was monitored and documented as follows: constantly at the central warehouse, once a day at the AKU warehouse, twice or more each day at the logistics center, and in the field (by the team leader) at least twice a day. Alternate power supply was available at all storage locales. Cooling equipment at each storage site consisted of cold rooms, a chest refrigerator at the logistics center, and cool boxes with frozen ice packs for the vaccination teams. Vaccine usage was recorded daily on a logistics form.

### Immunization campaign

The mass immunization campaign was planned and launched in a way that it would not disturb other regular local health programs. Vaccination took place from Monday through Sunday from 3 pm to 11 pm; this timing allowed the parent/guardian (usually a working male) flexibility to visit after the working hours. Each vaccination team was in charge of one cluster and was assigned to deliver one and only one vaccine code letter (C, M). The chance of breaking the code was reduced by explaining the purpose of blinding to the teams, rechecking by another person at the time of distribution and follow up visits by site supervisors to ensure the same code is being given in the cluster that is assigned after randomization. All efforts were made to ensure blinding.

Based on a pre-assigned schedule, vaccination teams visited each cluster from August 12^th ^to September 12^th^, 2003, to cover the target population of 21,059 children. Any child with fever > 37.5°C at the time of immunization or a female who was married, pregnant, and/or lactating was considered ineligible. Febrile individuals were provided with antipyretics and asked to return if the temperature subsided.

A vaccine record book (per cluster) containing a page-by-page alphabetical listing of all household members (based on a project census conducted 6 months earlier) was available to each team. Children were identified by their project identification (ID) card; if the card was not available, a computerized ID search system was used. The record book also documented the date of vaccination, eligibility, letter code of the vaccine, and presence or absence of an immediate AE. Team assistants (who belonged to the study area and have been working with AKU for at least one year) repeatedly visited the households in a specific cluster to re-invite and also to update household status such as migration, refusals, temporary absentees, and census duplications. The clusters where the vaccine coverage was less than 60% were visited again in the last 4 days of the campaign by re-establishing the vaccine post.

Vaccination AE data were obtained by direct observation of each vaccinee at each vaccination center for 30 minutes to detect immediate serious AE; by home visits (once/day, total of 3) in a cluster-based random sub-sample of 240 children (4 per cluster) to detect solicited AE; and by passive surveillance of un-solicited AE in the initial 30 days. Vaccination posts had basic emergency equipment and trained study staff to treat immediate severe AE (SAE); transportation to the AKU hospital was assured for SAEs. WHO guidelines on safe injection practices [17] were followed. Needlestick injuries were reported to the supervisor and treated in accordance with national guidelines. Disposal boxes for the safe disposal of sharps were provided to each team; the boxes were later incinerated at AKU hospital.

### Outcomes, data management, and statistical analysis

The primary outcome of the trial is an episode of fever during which S. Typhi is isolated from blood culture or fever ≥ 3 days and a positive serology-proven typhoid fever test (Widal test or Tubex or Typhidot-M) or fever ≥ 3 days and a positive Widal test or fever ≥ 24 hours and body temperature ≥ 38.5C and ≥ 1 of the following symptoms: headache, abdominal pain or constipation and a positive (Widal or Tubex or Typhidot-M) test.

The denominator for the incidence rate calculations will be the number of subjects at risk and it will be the primary denominator used to measure the outcomes.

Analysis of the surveillance data will be based on a fixed cohort approach and the incidence of typhoid fever in a two-year period will be calculated. Vaccine coverage was calculated on the basis of the vaccination record books and the proportion immunized from the target population. To assess logistics, the following were described and quantified: (1) resources, including personnel, needed for vaccine storage, transport, and delivery; (2) efficiency of vaccine storage, transport, and handling; and (3) safe vaccination practices, including vaccine administration and disposal of sharps.

Data were maintained with a FoxPro 6 (Microsoft, Redmond, WA USA) based data management system. Simple descriptive statistics such as frequencies, averages estimates, and proportions with standard deviation were calculated using SPSS version 10 (SPSS Inc., Cary, NC, USA). ArcView 8 (ESRI, USA) was used for geographical information system analysis.

## Results

### Vaccine coverage

In total, 12,830 (61%) children were vaccinated during the campaign. Of the remaining target population of 8,229 who did not receive vaccine, main reasons were emigration (2,824), refusal to participate (2,613), absence (1,811), ineligible (69), vaccinated in other programs (17), and incorrect census data (895) (table 1). The final coverage in the study sites was 74% with highest coverage in children aged 2–10 years (77%) and lower in those >10 years (67%). Overall coverage by gender and area was similar (table 2). Cluster vaccination varied from 37% to 81%, with median cluster coverage of 63% (figure 1). Second visit to the low coverage clusters in the last days (mop-up) increased the overall coverage from 70% to 74%.

**Table 1:** Vaccination coverage results from mass vaccination campaign in urban squatter settlements of Karachi, Pakistan – 2003

**Target population**	No. of individuals	%
**Not vaccinated**	**21,059**	
Migrated	2,824	13.4
Census programmatic error	895	4.2
**Total population eligible for vaccination**	**17,340**	
Vaccinated	12,830	74.0
Ineligible (fever/pregnancy/etc.)	69	0.4
Absent	1,811	10.4
Refused	2,613	15.1
Already received typhoid vaccine	17	0.1

**Table 2:** Demographic Data of study population present at the time of Mass Vaccination Campaign by area of residence in Karachi, Pakistan – 2003

				Hijrat			Sultanabad		
	**Total**	**Received vaccine**	**%**	**Total**	**Received vaccine**	**%**	**Total**	**Received vaccine**	**%**

**Total**	17,340	12,830	74	9,832	7,160	73	7,508	5,670	76
**Gender**									
Male	9,232	6,767	73	5,139	3,701	72	4,093	3,066	75
Female	8,108	6,063	75	4,693	3,459	74	3,415	2,604	76
**Age group, yrs**									
< 5	4,788	3,585	75	2,546	1,881	74	2,242	1,704	76
5 – 10	7,909	6,149	78	4,553	3,518	77	3,356	2,631	78
> 10	4,643	3,096	67	2,733	1,761	64	1,910	1,335	70

### Safety

There were 116 children with AE (<1%) of which 108 were detected by passive surveillance by the vaccination teams or health center staff. Of the 116 events, 53 were considered by the study physicians to be probably related to the vaccines. Among 139 persons surveyed three days after vaccination, 5 solicited AE were detected and none was considered a serious event. Three persons were hospitalized post immunization and were managed as an SAE until the DSMB and clinical monitor labeled them not to be related with the study vaccines. One child developed petchial heamorrhges and later was found out to be having a bleeding disorder. Another child was admitted with fever and was diagnosed as having culture proven typhoid. The third one had developed an injection abscess. The main adverse events reported included fever (48), local pain (56) and local swelling (15). No needle stick injuries were reported.

### Cold chain

There was no important deviation of the cold chain at any storage site. The central warehouse maintained the vaccine from +5°C to +6°C (mean +5.8°C); the AKU warehouse at +2°C to +8°C (mean 4.8°C), and the logistics center at +1°C to +12°C (mean 4.8°C). No vaccines were frozen. Alternate power supply was not needed at any locale.

The temperatures recorded at the vaccination posts were +3°C to +20°C (mean +4°C). These were maintained by 4 or 5 ice packs per cool box. The highest temperatures were observed during the busy hours when cool boxes were opened frequently. Temperatures were never above 8°C for more than 2 hours and were within the manufacturer's recommended guidelines. Thus, no vaccine had to be discarded because of temperature variation.

### Resources and supplies

On an average, 389 children were vaccinated per day and each vaccination team worked 7 hours a day for 33 days. A total of 12,837 vaccine doses were opened during the vaccination campaign. Seven doses were not used because the needle was injected in the vein.

## Discussion

The results of the program in two Karachi squatter settlements show that a large-scale vaccination program has good acceptance (74% vaccine coverage) and poses no major safety problems. The cold chain was maintained throughout the study in acceptable ranges. Thus, a mass vaccination campaign in squatter settlements is logistically feasible and safe.

Improved sanitation and food hygiene are the long-term solutions for reducing or eliminating typhoid fever [18] but these approaches are linked to socio-economic progress, which is slow in areas endemic for the disease. Hence large immunization schemes as public health measures are being recommended [9].

Some suggestions and plans for adoption based on our trial are considered below:

### Sample size

The targeted sample size could not be achieved due to refusals and non-response during the vaccination campaign. Since; the target population in the study setting was lower than expected. The effect of decreased coverage on the power of the study and hence on the result was obvious. Therefore in consultation with a team of statisticians the number of clusters was increased to 120 and the study was extended in another setting of Karachi with comparable socio-economic characteristics. In 2004 in a separate vaccination campaign we were able to vaccinate 14406 children of the similar age group from the remaining 60 clusters. In this way the targeted sample size was achieved. However it is very important that careful assessment of factors that affect response in needed in future trials to overcome problems that affect statistical power.

### Community involvement

First, the community mobilization strategy is pivotal. The information dissemination strategy, which was based on early stage involvement of community members with project personnel, provided frequent opportunities for clarifying misconceptions. This, in turn, brought about unprecedented community rapport and response. Community leaders took children to the vaccination posts when their mothers or female guardians were unable to do so and facilitated the vaccination of teenage girls. More intensive campaigns in areas with low vaccine uptake, areas that are politically and religiously unique from other clusters could increase coverage.

Although refusals rates were high in this vaccination campaign no direct resistance was seen. Some of the concerns mentioned included; 1) Why has this site been chosen for the trial, 2) Why are vaccines coded? 3) Why is the vaccine given for free? These concerns were dealt with by senior field staff. A senior person from the team would visit the household to answer questions. The questions that were asked for the first time were then added to our list of frequently asked questions and were discussed in the next interaction with the community.

Religious leaders have a significant influence on the intervention projects in developing countries, especially in Muslim majority populations. We interacted with them during the campaign after realizing the religious concerns of the people. The religious institutes such as mosques should be involved as part of community mobilization plan very early in the project.

Nevertheless we are aware the trial nature of the vaccination process could have impinged on the coverage results. Collectively those who refused to participate in the trial (15%) and those present and did not show-up (10%) make up a significant proportion of the target population (25%). The parents who refused to vaccinate their children were hesitant to give their children a vaccine with a code, despite detailed information was provided to them by the project staff. Out side trial conditions the refusal rate is therefore expected to be lower.

### Cost-effectiveness

Cost-effectiveness or cost-benefit of Vi PS for Pakistan is critical to convince decision-makers to finance public sector use of a new vaccine. Data from slums in New Delhi, India, indicate that typhoid fever is a disease with high economic consequences where the direct costs of the disease was more than US $100 per hospitalized typhoid fever episode. More than half of these costs come directly "out of pocket" rather than through government subsidies [19]. Another vaccine policy analysis in the area also suggests that a vaccine such as Vi PS would provide cost-saving to society [20]. Current results from this trial suggest the logistic feasibility, but it is understood that quantification and cost-estimation would be an important factor in terms of acceptance by the government.

### Implementation outside of EPI program

Vi PS vaccine, which does not induce immunological memory [4], is probably not a long-term vaccine option for Pakistan and other typhoid fever-endemic countries for that matter. Recently a Vi conjugate vaccine was shown to have high efficacy (89%) in Vietnamese children (ages 2–5 years) 46 months after immunization [21]. This vaccine potentially could provide long-term protection. Until the commercial availability of this vaccine, it will be important to consider alternative delivery scheme using the currently available typhoid vaccine. The trial has shown that approaching the population at risk – children aged 2 to 16 – through a mass immunization campaign was feasible.

### Timing of the campaign

The reported campaign took place at the end of school vacations when many households were visiting outside the study area. There were a significant (13%) proportion of migration between the census and the vaccine campaign (6-month period) but these findings should not be regarded as an impediment to the introduction of a Vi PS campaign.

## Conclusion

The introduction of new vaccines into developing countries, in particular to the most impoverished, requires basic research, clinical evaluations, epidemiological assessments, policy and economic research, establishment of production facilities, sound regulatory systems, procurement mechanisms, and distribution capabilities [22,23] In areas where typhoid fever is a serious and widespread problem, Vi PS vaccination appears to be the most promising available strategy for the control of typhoid fever in the short and mid term. The mass vaccination campaign described here showed that conducting a mass immunization outside of the EPI program and infrastructure is feasible and acceptable, and could potentially be implemented in a public health system.

## Competing interests

Vaccines were donated by GSK Biologicals, Rixensart Belgium.

## Authors' contributions

MIK was involved in the conduct of the mass vaccination campaign, supervision of data computerization and cleaning, analysis and drafting the manuscript RLO conduct of mass vaccination campaign, supervision of data computerization and cleaning – HBH conduct of mass vaccination campaign, supervision of data computerization and cleaning – SMS conduct of mass vaccination campaign, community mobilization and supervision of data computerization and cleaning – MAH coordinated the campaign activities in one site and supported the data management unit in data computerization – SBS was involved in the design and conduct of the trial – NSB coordinated campaign activities – SR supervised the data computerization and assisted in data analysis – MKP designed the data computerization software and assisted in data analysis – MA designed the data computerization software, helped in data analysis plan and assisted in data analysis – SMW coordinated the campaign and supervised the adverse event follow up – MJK supervised the surveillance for adverse events and coordinated the community mobilization – RAE was involved in design and development of procedure manuals – BI was involved in design and planning – CMG was involved in design and also planned the laboratory procedures – TP was involved in design and planning – AD designed the analysis plan, stratification and randomization – LvS was involved in planning, designing, and conduct of the study – CJA was involved in design, conduct, analysis, drafting the manuscript – JC conceived the study and was involved in design and conduct – SQN was involved in design and conduct – ZAB supervised the planning, design and implementation phase.

**Table 3:** Details of logistics used during the vaccination campaign in Karachi – Pakistan 2003

**Forms**	**First Aid Box**	**Basket**
Record book	Blood collection tubes	Resuscitator
Member list	Syringes (5 ml)	Spirit swabs
Household list	Tube stand	Cotton balls
ID card (undistributed)	Tourniquit	Disposable gloves
Informed consent	Butterfly needles	Disposal bags
Daily logistics	Inj Epinephrine	Lamp
Temperature chart	Inj Dexomethazone	**Additional supply**
Transfer sheet	Syringes (1 ml)	Umbrella
Progress sheet	Scissors	Fan
Tally sheet	Handiplast	Water cooler
Attendance sheet	Thermometer	**Others**
Immunogenicity	Extra needles	Safety box
Economics	Soap	Juices
IAE	**Shoulder bag**	
AE definition	Pen	
**Icebox**	Marker	
Icepacks	Notepad	
Replacement	Stamp pad	
Thermometer	Duct tape	

**Figure 2:**
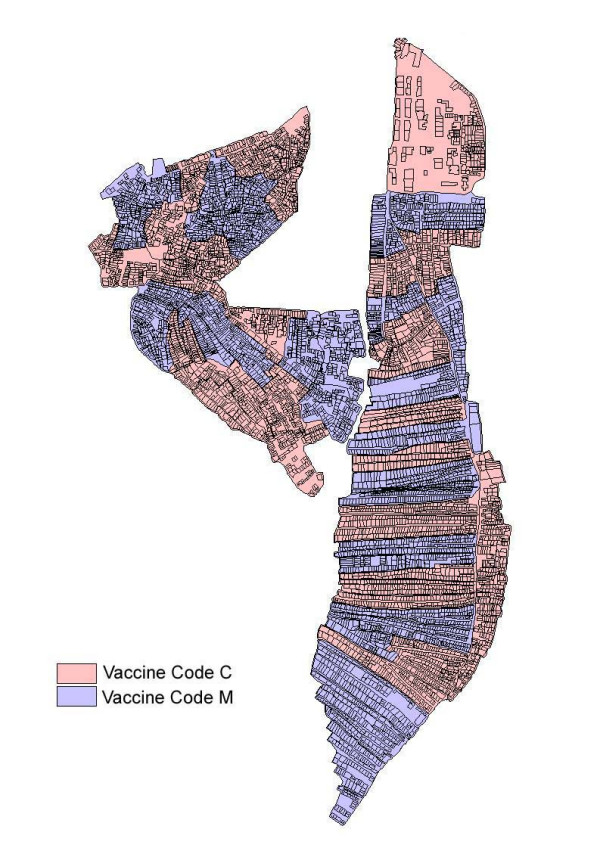
Cluster wise distribution of vaccine codes in Vi demonstration project Karachi – Pakistan 2003.

**Figure 3:**
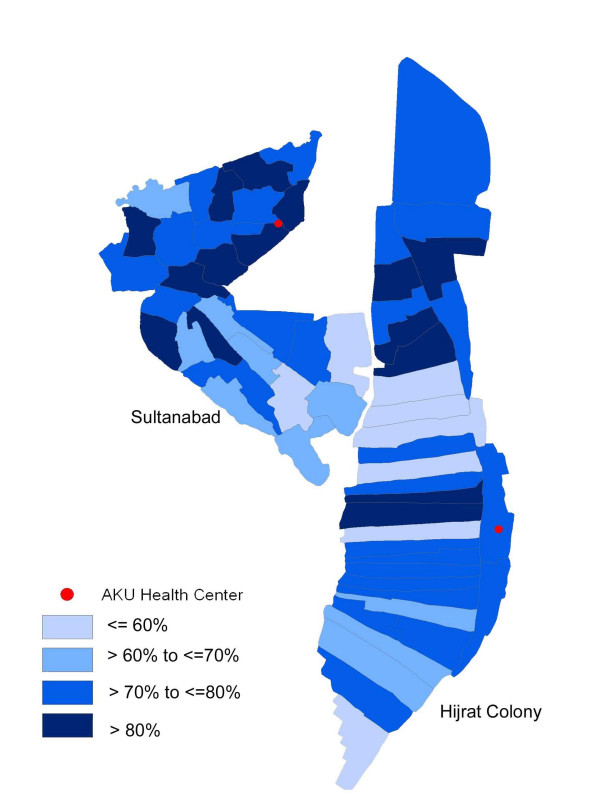
Cluster wise distribution of vaccination coverage during mass vaccination campaign in Vi demonstration project Karachi – Pakistan 2003.

## References

[B1] Bank W (2004). World Development Report 2004: Making services work for poor people.

[B2] Acosta CJ, Galindo CM, Ali M, Elyazeed RA, Ochiai RL, Danovaro-Holliday MC, Page AL, Thiem VD, Jin Y, Park JK, Lee H, Puri MK, Ivanoff B, Agtini MD, Soeharno R, Simanjuntak CH, Punjabi NH, Canh do G, Sur D, Nizami Q, Manna B, Bai-qing D, Anh DD, Honghui Y, Bhattacharya SK, Bhutta Z, Trach DD, Xu ZY, Pang T, Donner A, Clemens JD (2005). A multi-country cluster randomized controlled effectiveness evaluation to accelerate the introduction of Vi polysaccharide typhoid vaccine in developing countries in Asia: rationale and design. Trop Med Int Health.

[B3] Engels EA, Falagas ME, Lau J, Bennish ML (1998). Typhoid fever vaccines: a meta-analysis of studies on efficacy and toxicity. Bmj.

[B4] Hessel L, Debois H, Fletcher M, Dumas R (1999). Experience with Salmonella typhi Vi capsular polysaccharide vaccine. Eur J Clin Microbiol Infect Dis.

[B5] Robbins JD, Robbins JB (1984). Reexamination of the protective role of the capsular polysaccharide (Vi antigen) of Salmonella typhi. J Infect Dis.

[B6] Siddiqui FJ, Rabbani F, Hasan R, Nizami SQ, Bhutta ZA (2006). Typhoid fever in children: some epidemiological considerations from Karachi, Pakistan. Int J Infect Dis.

[B7] Bhutta ZA, Khan IA, Shadmani M (2000). Failure of short-course ceftriaxone chemotherapy for multidrug-resistant typhoid fever in children: a randomized controlled trial in Pakistan. Antimicrob Agents Chemother.

[B8] Bhutta ZA (1996). Impact of age and drug resistance on mortality in typhoid fever. Arch Dis Child.

[B9] WHO (2003). Background Document: Diagnosis, treatment and prevention of typhoid fever.

[B10] Levine MM, Orenstein SAPWA (1999). Typhoid fever vaccines. Vaccines.

[B11] WHO/UNICEF (2003). Review of National immunization coverage 1980 - 2002 Pakistan.

[B12] Ahmad N, Akhtar T, Roghani MT, Ilyas HM, Ahmad M (1999). Immunization coverage in three districts of North West Frontier Province (NWFP). J Pak Med Assoc.

[B13] Shaikh I, Omair A, Inam SN, Safdar S, Kazmi T, Anjum Q (2003). National Polio Day campaign in a squatter settlement through medical students. J Pak Med Assoc.

[B14] Khan Z (1993). Immunisation and infant mortality in Pakistan. Pak Dev Rev.

[B15] Ahmad KAKLHRBBZA (2000). A twelve year clincal experience with paediatric salmonellosis from an endemic population in Karachi: Argentina..

[B16] Hayes RJ, Bennett S (1999). Simple sample size calculation for cluster-randomized trials. Int J Epidemiol.

[B17] WHO (2000). Supplementary information on vaccine safety. Part I: Field issues.

[B18] Parry CM, Hien TT, Dougan G, White NJ, Farrar JJ (2002). Typhoid fever. N Engl J Med.

[B19] Bahl R, Sinha A, Poulos C, Whittington D, Sazawal S, Kumar R, Mahalanabis D, Acosta CJ, Clemens JD, Bhan MK (2004). Costs of illness due to typhoid fever in an Indian urban slum community: implications for vaccination policy. J Health Popul Nutr.

[B20] Poulos C, Bahl R, Whittington D, Bhan MK, Clemens JD, Acosta CJ (2004). A cost-benefit analysis of typhoid fever immunization programmes in an Indian urban slum community. J Health Popul Nutr.

[B21] Mai NL, Phan VB, Vo AH, Tran CT, Lin FY, Bryla DA, Chu C, Schiloach J, Robbins JB, Schneerson R, Szu SC (2003). Persistent efficacy of Vi conjugate vaccine against typhoid fever in young children. N Engl J Med.

[B22] Mahoney RT, Ramachandran S, Xu Z (2000). The introduction of new vaccines into developing countries II. Vaccine financing. Vaccine.

[B23] Mahoney RT, Maynard JE (1999). The introduction of new vaccines into developing countries. Vaccine.

